# The Micro-Immunotherapy Medicine 2LARTH® Reduces Inflammation and Symptoms of Rheumatoid Arthritis *In Vivo*

**DOI:** 10.1155/2020/1594573

**Published:** 2020-01-23

**Authors:** Ilaria Floris, Víctor García-González, Belen Palomares, Kurt Appel, Beatrice Lejeune

**Affiliations:** ^1^Preclinical & Clinical Development and Regulatory Affairs, Labo'Life France, 1 Rue François Bruneau, 44000 Nantes, France; ^2^Innohealth Group, Madrid, Spain; ^3^Instituto Maimónides de Investigación Biomédica de Córdoba (IMIBIC), Córdoba, Spain; ^4^Departamento de Biología Celular, Fisiología e Inmunología, Universidad de Córdoba, Córdoba, Spain; ^5^Hospital Universitario Reina Sofía, Córdoba, Spain; ^6^VivaCell Biotechnology GmbH, Denzlingen 79211, Germany; ^7^Labo'Life Belgium, Parc Scientifique Crealys, Rue Camille Hubert 11, 5032 Gembloux, Belgium

## Abstract

**Background:**

Rheumatoid arthritis (RA) is a chronic inflammatory joint disease, which can cause cartilage and bone damages as well as pain and disability. In order to prevent disease progression, reduce pain, and major symptoms of RA, one good strategy consists in targeting proinflammatory cytokines that have the key role in the vicious circle of synovial inflammation and pain. The micro-immunotherapy medicine (MIM) 2LARTH® targets cytokines involved in inflammation.

**Aim:**

The aim of the study is to evaluate the effect of the MIM compared to vehicle in an *in vivo* model of RA, induced in mice after immunization with articular bovine type II collagen.

**Methods:**

Vehicle and MIM were dissolved in pure water (1 capsule in 100 ml) and 100 *µ*l was given by gavage daily for 14 days. To evaluate the severity of arthritis, wrist and ankle thickness was determined, paw edema was measured, and a clinical score from 0 to 4 was established. Furthermore, histological analysis was performed. To evaluate systemic inflammation, circulating levels of IL-1*β* and TNF-*α* were measured by ELISA.

**Results:**

Ankle thickness was found to be significantly reduced in MIM-treated mice compared to vehicle-treated mice (*P* < 0.05) and compared to untreated me (*P* < 0.05) and compared to untreated me (*P* < 0.05) and compared to untreated me (*β* and TNF-*α* were measured by ELISA. *P* < 0.05) and compared to untreated me (

**Conclusion:**

The results indicate that the tested medicine reduces inflammation, histological, and clinical signs of RA in a CIA model.

## 1. Introduction

Rheumatoid arthritis (RA) is an immune-mediated, systemic inflammatory disease that affects mainly the synovial joints, characterized by intraarticular inflammation, synovial hyperplasia, and progressive degradation of cartilage and bone. RA is one of the most prevalent chronic inflammatory diseases; its prevalence is around 1% of the population, and the disease is more frequent (~2 : 1) and more severe in women than in men [1].

Genomic studies have identified more than a hundred loci associated with RA risk, mostly implicated in immune mechanisms and chronic inflammatory diseases. In particular, the human leukocyte antigen (HLA) system is strongly linked to susceptibility of RA [2]. Furthermore, some HLA polymorphisms are associated to a more aggressive form of RA and higher mortality [3].

Joint inflammation in RA is at the apex of clinical events: pain, swelling, and stiffness, development of adhesions, erosion of joint surfaces, bone resorption, loss of function, and joint deformation [4].

The major therapeutic goal in RA consists in reversing the chronic inflammation. There have been clear advances in the pharmacological management of RA over the past decade, nevertheless many patients still do not tolerate or do not respond well to the available therapies. The current approach consists of tight control of disease activity through disease-modifying anti-rheumatic drug (DMARD). This therapy targets the inflammation and is aimed at blocking disease progression and joint damage. Both the American College of Rheumatology and the European League against Rheumatism recommend that DMARDs treatment should start as soon as a diagnosis has been made [5]. However, it is not easy because no diagnostic criteria exist for RA. Traditional nonsteroidal anti-inflammatory drugs are also widely used to reduce pain and stiffness, yet those drugs do not interfere with joint damage and, as glucocorticoids, offer rapid symptomatic relief, but are associated with serious long-term side effects [6, 7].

Despite the improvements made in the past two decades in management of RA symptoms, many issues remain to be addressed. Mainly, we cannot predict optimal responses neither toxic, nor side effects for a given treatment.

Micro-immunotherapy (MI) is a therapeutic approach which can be used alone or in association with other therapies to offer clinical benefits without side effects. MI uses molecules at low doses (LD) and ultralow doses (ULD) impregnated on lactose-saccharose pillules for oromucosal administration, to target the immune system and regulate immune responses in diseases. The active substances used in MI medicines (MIM) are cytokines, hormones, growth factors, neuropeptides, nucleic acids, and specific nucleic acids (SNAs®). SNAs® consist of single strands of DNA molecules, ranging from 16 to 34 bases, designed to target specifically 1 or more genes (European Patent: EP0670164B1).

2LARTH® is a MIM notified as a homeopathic medicine under notification number 1507CH36 F1 by the Federal Agency for Medicines and Health Products in Belgium. It has been developed to counteract the over-expression of interleukin (IL)-1*β* and tumor necrosis factor (TNF)-*α*, once the inflammatory cascade is activated and the chronic inflammatory status is established. Furthermore, the tested MIM aims at targeting HLA system and IL-2. The anti-inflammatory effect of the medicine has been demonstrated *in vitro* on human enriched monocytes exposed to lipopolysaccharide [8].

To evaluate its efficacy *in vivo*, authors planned a research project in collagen-induced arthritis (CIA). CIA is induced in mice with articular bovine type II collagen (CII) and the resulting disease is essentially mediated by an autoimmune response. CIA is commonly used to study *in vivo* the pathological mechanisms of RA and to evaluate new antiarthritic drugs as it shares clinical, histological, and immunological features of human RA [9, 10].

The sublingual administration is the mode of administration of MIM, including for the tested MIM. Because rodents have their buccal mucosa keratinized, pillules were dissolved in pure water, then administered orally by gavage. The oral route performed by gavage is convenient because it allows the use of lower doses, it minimizes side effects, and may provide enhanced efficacy [11]. Indeed, the gastrointestinal tract contains innate immune system cells (macrophages, monocytes, neutrophils, and dendritic cells), and cells of the adaptive immune system (T and B lymphocytes and their secreted mediators, cytokines, and chemokines). Organized lymphoid structures are present in the gut: small lymphoid nodules in the upper small intestine, and large organized aggregates of lymphoid tissue (Peyer's patches) in the ileum [12]. Other studies demonstrated that orally administered peptides may be detected by immune cells present in the intestinal lumen, and induce a specific immune response [13, 14].

The aim of the study is to evaluate *in vivo* the efficacy of MIM in treating clinical signs and chronic inflammation of arthritis, in a well-known animal model of RA.

## 2. Materials and Methods

### 2.1. Materials

The active substances present in the tested MIM composition are: IL-1*β*, TNF-*α*, IL-2, SNAs® targeting genomic regions, and transcripts of HLA class I, class II, and human IL-2. Each active substance is singularly prepared to obtain specific LD or ULD, following a “Serial Kinetic Process” (SKP), consisting of a 1/100 dilution process followed by vertical shaking. SKP is reproduced a defined number of times; number that is reported in composition as number of Centesimal Hahnemannian dilutions (CH).

The composition of the tested medicine is as follows: IL-1*β* at 10 CH, or at 17 CH, TNF-*α* at 10 CH, or at 17 CH, IL-2 at 10 CH, or at 12 CH, SNA-HLA-I at 10 CH, or at 16 CH, SNA-HLA-II at 10 CH, or at 16 CH, and SNA-ARTH at 10 CH, or at 16 CH. The excipient consists of lactose-sucrose pillules, also called globules (about 380 mg per capsule), which were impregnated with ethanolic preparation of active substances, at LD or ULD.

The tested MI medicine is a sequential drug and the composition of each capsule is specific. Indeed, capsules in blister are numbered from 1 to 10 and the intake should respect the sequential order.

The vehicle capsules used as controls contain lactose-saccharose pillules impregnated with the ethanolic preparation lacking active substances.

### 2.2. Animal Care and CIA Induction

DBA/1 mice (male 8 weeks-old) were purchased from Janvier Labs (France). All animals were kept on a 12 h light-dark cycle, at a controlled temperature (23 ± 1°C) and 40–50% relative humidity with free access to standard food and water. Experimental procedure was performed under the guidelines for care and use of animals specified by the Animal Research Ethic Committee of Córdoba University (Spain).

CIA was induced in 32 mice, while 6 mice were let as a control group. Briefly, this is the protocol used for mice immunization:

- Bovine collagen nasal septum type II (CII) (C7806, Sigma-Aldrich) was dissolved at 2 mg/ml in 10 mM acetic acid by stirring overnight at 4°C. CII was kept cold while being dissolved, to prevent CII denaturation.
- Heat-killed *Mycobacterium tuberculosis* (H37RA) (231141, BD) was combined with incomplete Freund's adjuvant (F5506, Sigma-Aldrich) at a 4 mg/ml final concentration.
- CII was emulsified in an equal volume (1 : 1) of CFA just prior to immunization using a high-speed homogenizer (Ultraturrax T8, IKA Labortechnik). Collagen was kept cold throughout the emulsification.

**Figure 1:**
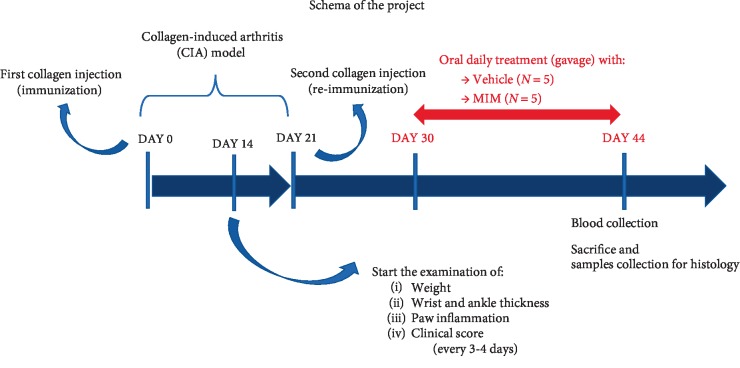
Experimental schema of the *in vivo* study.

During the phase of mice immunization, animals were awake and physically restrained. DBA/1 mice (8 weeks-old) were immunized with 100 *µ*l of CII/CFA emulsion by an intradermal injection at the base of the tail. At day 0 mice received the first immunization, then at day 21 the animals were reimmunized with a booster injection of the same composition and volume and at the same site (Figure 1). Among the 32 mice, only 15 showed clear evidences of disease induction before starting the treatments. Hence, mice with CIA were redistributed in three groups: CIA (*n* = 5), CIA + vehicle (*n* = 5), and CIA + MIM (*n* = 5).

### 2.3. Treatment

Starting from day 30 until day 44, animals were treated with vehicle (CIA + vehicle) or MIM (CIA + MIM), as represented in Figure 1. Both types of granules were dissolved in pure water (the content of 1 capsule in 100 ml) and 100 *µ*l was given daily for 14 days by gavage starting from day 30. The daily dose of sugar corresponds to 0.380 mg.

The order of administration respected the MIM sequentiality and the order from 0 to 10 indicated on the blister.

### 2.4. Clinical Evaluation of Arthritis Signs

To evaluate wrist and ankle inflammation, thickness (mm) was measured every 3-4 days using a Vernier caliper.

Every 3-4 days, paw edema was also measured using a plethysmometer (LE7500, Panlab). Basically, the water displacement produced by the immersion of the animal paw in the measuring tube is reflected into the second tube, inducing a change in the conductance between the two electrodes which generates an output signal indicating the volume displacement measured.

The scoring system for subjective evaluation of arthritis severity used the following scale: 0 = normal; 1 = detectable swelling in one joint or toe; 2 = swelling in two types of toes or joints but not entire paw inflamed; 3 = entire paw inflamed and swollen; 4 = severe swelling in the entire paw or ankylosed.

### 2.5. Histological Analysis

At 44 days, mice were sacrificed and the knee and paws tissues were extracted and prepared for histological analysis. Histopathology studies were performed in 3 representative animals of each group.

The joints (knee and paws) were fixed in 4% paraformaldehyde for 2 days and then decalcified in 7% nitric acid for 5 days. After decalcification, samples were processed for light microscopy; these were dehydrated in alcohol and embedded in paraffin. Sagittal serial sections were cut (5 *µ*m) and stained with hematoxylin-eosin (H&E) and Safranin-O.

#### 2.5.1. H&E Staining

To determine whether the treatment was effective, the joints were examined for the presence or absence of synovitis, pannus formation, cartilage loss, bone erosions, and disruption of joint architecture. The histologic slides stained with H&E were scored from 0 to 4 according to the following gradation of arthritis: 0 = normal ankle joint; 1 = normal synovium with occasional mononuclear cells; 2 = definite arthritis, a few layers of flat to rounded synovial lining cells and scattered mononuclear cells; 3 = clear hyperplasia of the synovium with three or more layers of loosely arranged lining cells and dense infiltration with mononuclear cells; 4 = severe synovitis with pannus and erosions of articular cartilage and subchondral bone. Images were taken using a Leica DM2000 microscope and Leica MC190 camera.

#### 2.5.2. Safranin-O Staining

Sagittal sections were deparaffinized in xylol, hydrated with decrescent concentrations of ethanol (100%, 96%, and 80%), and stained sequentially with Weigerts iron hematoxylin, 0.002% fast green, 1% acetic acid, and 1% Safranin-O, washed in running tap water, dehydrated by crescent concentrations of ethanol (80%, 96%, and 100%), incubated in xylol, and finally mounted with Eukitt mounting medium. Images were taken using a Leica DM2000 microscope and Leica MC190 camera. The cartilage area was determined in pixel using image *J* software.

### 2.6. Enzyme-Linked Immunosorbent Assay

Blood was collected at the end of treatment and plasma was obtained by centrifuging the samples in heparinized tubes (BD Vacutainer Heparin tubes) at 2000 × *g* for 20 min.

The expression of circulating IL-1*β* and TNF-*α* was measured by quantitative sandwich enzyme immunoassay technique according to the instructions of the manufacturers (R&D systems). Briefly, the technique employs the monoclonal antibody specific for mouse protein of interest (TNF-*α*, or IL-1*β*). Antibody has been precoated onto a microplate. Standards, control, and samples are pipetted into the wells and the immobilized antibody binds specific proteins such as TNF-*α*, or IL-1*β*. After washing away any unbound substances, an enzyme-linked polyclonal antibody specific for mouse protein of interest is added to the wells. Following washing to remove any unbound antibody-enzyme reagent, a substrate solution is added to the wells. The enzyme reaction yields a blue product that turns yellow when the stop solution is added. The intensity of the color measured is proportional to the amount of protein bound in the initial step. The sample values are then read off the standard curve.

### 2.7. Statistical Analysis

Data are expressed as mean ± SEM. To test whether the mean values were significantly different between groups, we used one-way ANOVA test followed by Tukey's post hoc test. To analyze differences between two groups, unpaired *T* test was used. The *p* value ≤0.05 was considered statistically significant. Statistical analysis was performed using GraphPad Prism version 8 (GraphPad, San Diego, CA, USA).

## 3. Results

### 3.1. Body Weight Measurement

All mice with collagen-induced arthritis, CIA, CIA + vehicle, and CIA + MIM, showed slight reduction in body weight compared to control mice. No significant changes have been found between mice which were treated with vehicle and MIM (data not shown).

### 3.2. Wrist and Ankle Thickness Determination

**Figure 2:**
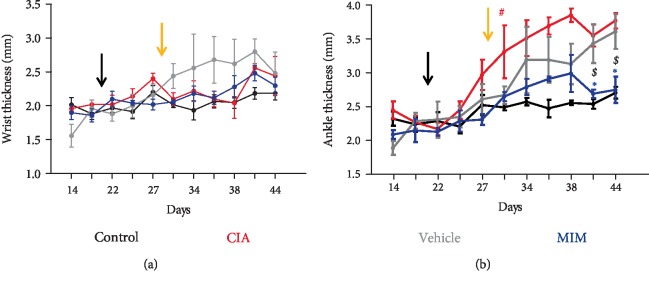
Variations in wrist and ankle thickness. (a) Wrist and (b) ankle thickness (mm) were measured every 3-4 days using a caliper. The black arrow indicates the reimmunization day, while the yellow arrow the beginning of the treatment with vehicle or MIM in their respective group. ^∗^*P* < 0.05 vs. vehicle-treated group; ^$^*P* < 0.01 vs. untreated CIA group; ^#^*P* < 0.05 vs. control group.

The results of the wrist thickness measurement did not show significant variations between the different experimental groups during the treatment (Figure 2(a)). However, mice groups that developed CIA showed an increase in ankle thickness starting from day 31 (*P* < 0.05 vs control). After 11–14 days of treatment, the ankle thickness was found to be significantly reduced in MIM-treated mice in comparison with vehicle -treated mice (*P* < 0.05) and in comparison with CIA untreated mice (*P* < 0.01) (Figure 2(b)).

### 3.3. Evaluation of Hind Limbs Edema and Severity Score

**Figure 3:**
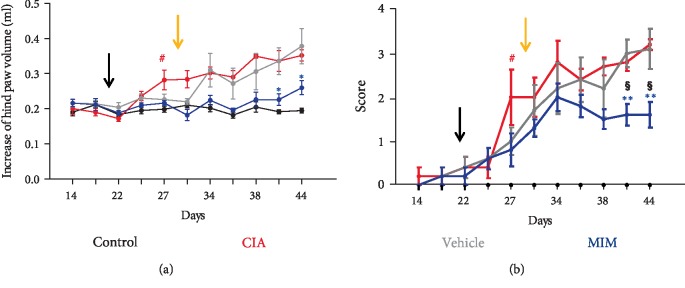
Variations in paw edema and clinical score. (a) Paw edema was measured as an increase in volume (ml), every 3-4 days using a plethysmometer. The black arrow indicates the reimmunization day, while the yellow arrow the beginning of the treatments. ^∗^*P* ≤ 0.05 vs. vehicle-treated group; ^#^*P* < 0.05 vs. control group. (b) Clinical score was attributed every 3-4 days for subjective evaluation of arthritis severity. The black arrow indicates the reimmunization day, while the orange arrow the beginning of the treatments. ^∗∗^*P* < 0.01 vs. vehicle-treated group; ^§^*P* < 0.01 vs. untreated CIA group; ^#^*P* < 0.05 vs. control group.

CIA and CIA + vehicle groups had a progressive increase in inflammation in the hind paw after the booster injection administered at day 21, in accordance with the induction of arthritis. Nevertheless, after 11–14 days of treatment, the paw edema was lower in mice treated with tested MIM than vehicle (*P* < 0.05), or untreated CIA mice (*P* < 0.05) (Figure 3(a)). The clinical score attributed at the end of treatment to active pillules-treated mice is lower than score of vehicle-treated mice (*P* < 0.01) or untreated CIA mice (*P* < 0.01), swelling was observed only in some toes or joints, but not the entire paw was inflamed. (Figure 3(b)). MIM-treated mice showed improvement of arthritis signs throughout the study, significant at the end of treatment (day 41 and 44).

### 3.4. Histological Analysis of the Hind Paws

**Figure 4:**
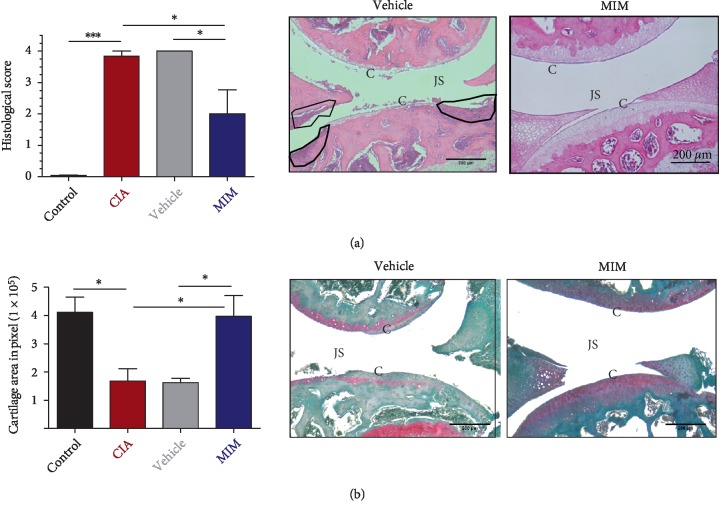
Histological examination of joints. (a) Histological score was determined based on arthritis severity observed in the sections of knee joints stained with H&E. (b) Quantification of cartilage area in pixel and representative histologic sections of knee joints of three animals per group stained with Safranin-O. The stained cartilage appeared red in color. Representative histologic sections for CIA + vehicle and CIA + MIM are shown next to each graph. More representative histologic sections are shown in Supplementary Materials, see Figures [Supplementary-material supplementary-material-1] and [Supplementary-material supplementary-material-1]. C = cartilage layer; JS = joint space; black circles indicate inflammation and synovial hyperplasia (pannus formation). Bars in graphs represent mean ± SEM of 3 mice per group. ^∗^*P* < 0.05, ^∗∗∗^*P* < 0.001.

The H&E staining of normal mice knee joints showed no inflammatory symptoms. On the other hand, the knee joint histology of CIA and CIA + vehicle groups presented pathological features of arthritis such as infiltration of lymphocytes, synovial hyperplasia, pannus, and articular cartilage loss with connective tissue replacement. In CIA + MIM mice, inflammation and joint destruction are lower in comparison to arthritic mice. In fact, the synovial membrane in the joints was almost like normal synovium, with few signs of synovial hyperplasia or other characteristics of inflammation. According to the score mentioned in materials and methods section for quantification of arthritis in the H&E samples, MIM-treated mice had a lower score than mice treated with vehicle (*P* < 0.05), and lower than untreated CIA mice (*P* < 0.05) (Figure 4(a), [Supplementary-material supplementary-material-1] in Supplementary Materials).

Safranin-O staining results showed an evident reduction of cartilage in the knee joint of untreated CIA mice (*P* = 0.01), and vehicle treated mice (*P* = 0.01), while the aspect of cartilage in mice treated with MIM is quite similar to the control group (Figure 4(b), [Supplementary-material supplementary-material-1] in Supplementary Materials). The subsequent quantification shows that the mice which received MIM present clearly reduced morphological alterations characteristic of arthritis, corroborating the previous H&E results.

### 3.5. Determination of Proinflammatory Cytokines Levels in Plasma

**Figure 5:**
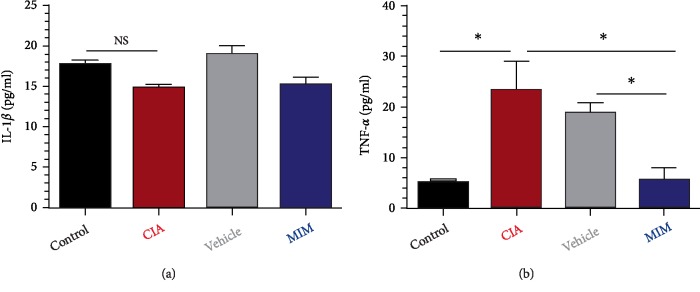
Inflammatory cytokines levels in plasma analyzed by ELISA. The optical density of each sample was measured at 450 nm, using the microplate reader TECAN. (a) IL-1-*β* levels (pg/ml), and (b) TNF-*α* levels (pg/ml) are represented in graphs, ^∗^*P* < 0.05.

No significant changes were found in IL-1*β* plasma levels between untreated CIA and controls (Figure 5(a)). In contrast, plasma levels of TNF-*α* increased significantly in the CIA group (*P* = 0.01, vs. control), but were significantly reduced in the mice treated with tested MIM, *P* < 0.05 vs. CIA groups (Figure 5(b)).

## 4. Discussion

CIA is one of the most useful models to study chronic inflammation occurring during RA, indeed autoreactivity of T and B cells, joint inflammation, cartilage, and bone damage, are similar to the human disease. In CIA model, TNF-*α*, IL-1*β*, and IL-6 play a key role in the early phase of the disease and continue to be important during the transition to the chronic phase [15]. TNF-*α*, IL-1*β*, and IL-6 are also osteolytic cytokines that are involved in the destruction of bone and joints in RA as implicated in promoting the expression of receptor activator of nuclear factor kappa-B ligand or RANKL in synovial fibroblasts, in the differentiation of osteoclasts and also in the induction of matrix metalloproteinase production [16, 17].

The gene inactivation of those three cytokines can partially/totally protect against RA [18–20]. IL-2 is also implicated in the early onset of RA [21]. The transition from acute to chronic inflammation is decisive in the shift of the immune system responses from defensive to tissue-damaging responses.

Cytokines can directly and indirectly generate pain. The direct effects on nociceptive neurons have been explained by: (i) the presence of nociceptive sensory neurons which express receptors for cytokines; (ii) *in vitro* studies on primary sensory neurons demonstrating that cytokines can change the excitability of neurons, modify ion currents, and regulate molecules involved in nociception; (iii) the injection of cytokines like TNF-*α* or IL-1*β* into normal tissue produces pain and enhances the responsiveness of nociceptive sensory fibers [22].

Hence, cytokines contribute to pain indirectly through the generation of inflammation and the production of prostaglandins and other regulators associated with pain. Targeting those cytokines can reduce inflammation-associated pain [22–24]. In particular, the neutralization of TNF-*α* rapidly reduced pain and inflammatory hyperalgesia in the absence of any other antinociceptive drugs. The effect of TNF-*α* on nerve fibers can be more easily reversed than the effect of other pain-related cytokines; indeed, each cytokine has a specific potential to induce chronic pain states [22].

The tested MIM aimed at inhibiting the mentioned cytokines involved in RA during early and chronic phases. MIM consists of lactose-saccharose pillules impregnated with LD or ULD of 3 human recombinant proinflammatory cytokines (IL-1*β*, TNF-*α*, and IL-2), LD or ULD of SNA® targeting genomic regions and transcripts of molecules belonging to HLA class I and class II, LD or ULD of SNA® targeting human IL-2.

*In Vitro* studies demonstrated the specific anti-inflammatory effect of tested MIM on human primary enriched monocytes, activated with lipopolysaccharide: MIM treated cells secreted lower levels of IL-1*β*, TNF-*α*, and IL-6 than vehicle and untreated cells [8].

The *in vivo* study described here demonstrates the efficacy of the tested MIM to treat CIA, starting from the early phase of the disease. The mice were daily treated with vehicle or MIM, following the sequential administration indicated on the blister. CIA + MIM mice were less impacted by ankle inflammation (Figure 2(b)) and paw edema (Figure 3(a)) compared to CIA + vehicle and untreated mice. In line with those results, clinical score attributed to MIM treated mice was found to be lower compared to vehicle control mice, only some toes or joints were inflamed, but not the entire paw as for CIA and CIA + vehicle groups (Figure 3(b)).

The most significant characteristic of RA is the chronic and intensive inflammation that is out of control. Other characteristics are the joint swelling and the leucocytes infiltration, which both reflect synovial membrane inflammation consequent to immune activation.

Histological analysis confirmed the presence of inflammation, immune infiltration, and joint destruction in CIA mice, while the aspect of joints in CIA + MIM group was almost like the one observed in control mice, with only few signs of synovial hyperplasia and inflammation. (Figure 4(a), [Supplementary-material supplementary-material-1]). The joint cartilage destruction detected in Safranin-O staining sections of CIA and CIA + vehicle groups, was not present in MIM-treated mice, in which the aspect of cartilage was more similar to those of the control group (Figure 4(b), [Supplementary-material supplementary-material-1]).

To check the impact on systemic levels of targeted cytokines, plasmatic levels of IL-1*β*. and TNF-*α* were analyzed at day 44, before the animal sacrifice. IL-1*β* levels were not found to be increased in CIA mice compared to control mice, and no difference were found between treated and CIA group, as reported in another animal [25] and human studies [26] (Figure 5(a)), while plasmatic levels of TNF-*α* increased significantly in CIA group compared to control group, and were found to be lowered in MIM-treated mice in comparison to the rest of the mice with CIA (*P* < 0.05 vs. CIA and vs vehicle groups) (Figure 5(b)).

A crosstalk between the mucosal surfaces (buccal, intestinal, nasal, etc.) and the systemic immune system exists [27, 28] and oral immunotherapy, including MI, use this concept to target immune system imbalances.

**Figure 6:**
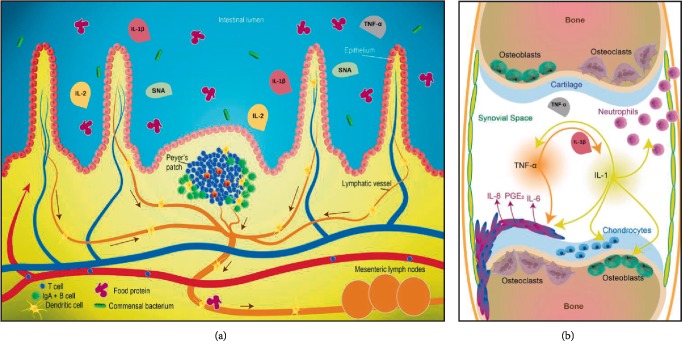
Mode of action of tested MIM. (a) Oral administered MIM can interact with cells of the innate and adaptive immune system present in the gut, reducing systemic inflammation and in turn, local inflammation of the synovial compartment. (b) RA is characterized by the presence of cytokines in the synovial compartment, fibroblasts assume an aggressive inflammatory phenotype, and chondrocytes enhance their catabolism promoting articular destruction. The tested MIM aimed at reducing levels of proinflammatory cytokines involved in pain and clinical signs of RA.

The intestinal Peyer's patches are connected with lymphoid tissues to coordinate immune responses to pathogens in the gut, as well as to maintain food tolerance, bacterial, and virus defenses. Immune cells can be found inside the matrix of Peyer's patches, including T and B cells, macrophages, dendritic cells, and specialized phagocytic cells known as M cells [12]. The hypothesized mode of action of the tested MIM can be explained with the involvement of those immune cells and structures present in the gut, which are able to communicate with the systemic immune system and in turn, with the cells present in the inflammatory milieu of the synovial compartment (Figure 6).

At the molecular level, the mode of action of ULD and MIM, might be explained with the implication of the hormesis [8]. The concept of hormesis, which was introduced for the first time 130 years ago by Schulz, is now becoming prominent in toxicology, pharmacology, and in many biomedical areas. Indeed, hormetic dose responses are widely present in microbial, plant, animal models, and humans [29].

## 5. Conclusion

The results obtained in this study showed that the oral administration of MIM effectively reduces the clinical score and the degree of edema, and inflammation caused by collagen-induced arthritis in DBA/1 mice. Furthermore, the treatment attenuates the CIA pathological features since it was able to reduce the infiltration of inflammatory cells, synovium hyperplasia, and cartilage loss in comparison to the vehicle treatment and untreated CIA groups. In addition, plasmatic levels of the proinflammatory cytokine TNF-*α* were decreased by the tested MIM.

In conclusion, we demonstrated that the tested medicine reduces the signs of arthritis and can be used in the pharmacological management of RA. Due to the chronic nature of RA, the pain and the disabilities related with the disease and, considering the side effects associated with the actual clinical treatments of RA, the tested MIM is a promising therapy, compatible with other therapeutic agents, and safe because no toxicity is caused by MIM. Additional preclinical studies are needed to fully understand the mode of action of the tested medicine, as well as clinical studies to validate the efficacy appreciated in CIA mice model also in human.

## Data Availability

The raw datasets analyzed during the current study are available from the corresponding author on reasonable request.

## Abbreviations

RA: Rheumatoid arthritis
HLA: Human leukocyte antigen
DMARD: Disease-modifying anti-rheumatic drug
MI: Micro-immunotherapy
LD: Low doses
ULD: Ultra-low doses
MIM: Micro-immunotherapy medicine
SNA®: Specific nucleic acids
IL: Interleukin
TNF-*α*: Tumor necrosis factor-*α*
CIA: Collagen-induced arthritis
CII: Type II collagen
SKP: Serial kinetic process
CH: Centesimal Hahnemannian dilutions
CFA: Complete Freund's adjuvant
H&E: Hematoxylin and eosin
ELISA: Enzyme linked immunosorbent assay.

## Supplementary Materials

Supplementary Material Figure S1. Representative histologic sections of knee joints of three animals per group stained with H&E from normal (1a–3c), CIA (4a–6c), vehicle (7a–9c) and MIM-treated mice (10a–12c) are shown. The magnification of the images in the first row was taken at 4x, whereas in the second and third row were taken at 10x. Black circles indicate inflammation and synovial hyperplasia. Green circles show articular cartilage loss. T=Tibia; F=Femur; C=Cartilage layer; JS=Joint space; E=Epiphyseal growth plate. Figure S2. Representative histologic sections of knee joints of three animals per group stained with Safranin-O from normal (1–3), CIA (4–6), placebo-treated (7–9) and 2LARTH-treated mice (10–12) are shown. The magnification of the images was taken at 4x and 10x. The areas with cartilage appear red colour stained.
