# Supplementary material for: The Micro-Immunotherapy Medicine 2LARTH® Reduces Inflammation and Symptoms of Rheumatoid Arthritis In Vivo
Source: Int J Rheumatol. 2020 Jan 23;2020:1594573. doi: 10.1155/2020/1594573 (PMC7064837; doi:10.1155/2020/1594573)
Supplement: Supplementary Material — Figure S1. Representative histologic sections of knee joints of three animals per group stained with H&E from normal (1a–3c), CIA (4a–6c), vehicle (7a–9c) and MIM-treated mice (10a–12c) are shown. The magnification of the images in the first row was taken at 4x, whereas in the second and third row were taken at 10x. Black circles indicate inflammation and synovial hyperplasia. Green circles show articular cartilage loss. T=Tibia; F=Femur; C=Cartilage layer; JS=Joint space; E=Epiphyseal growth plate. Figure S2. Representative histologic sections of knee joints of three animals per group stained with Safranin-O from normal (1–3), CIA (4–6), placebo-treated (7–9) and 2LARTH-treated mice (10–12) are shown. The magnification of the images was taken at 4x and 10x. The areas with cartilage appear red colour stained. [file 1594573.f1.docx]

**Supplementary Materials**


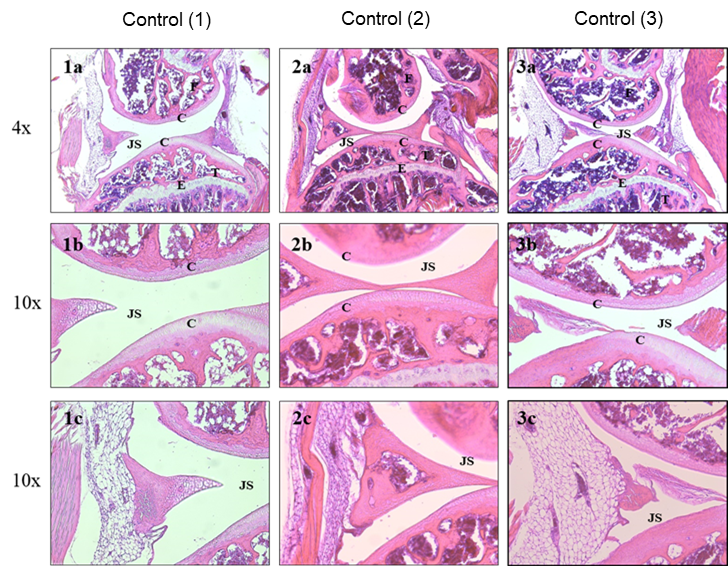


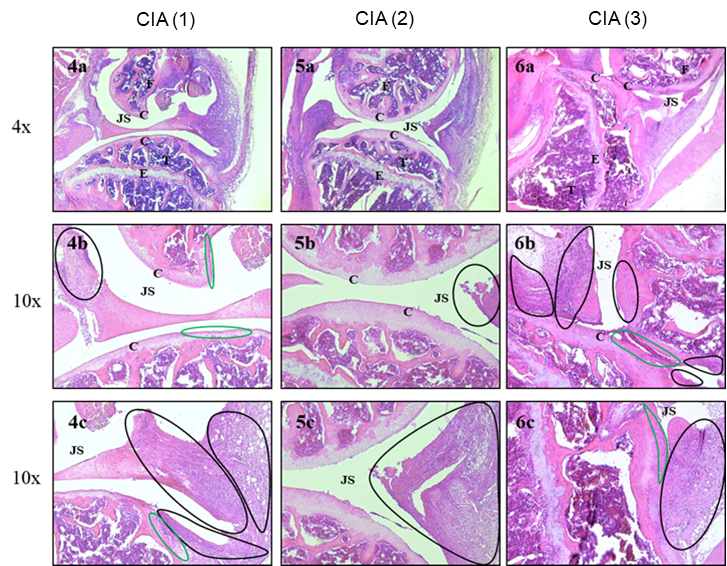


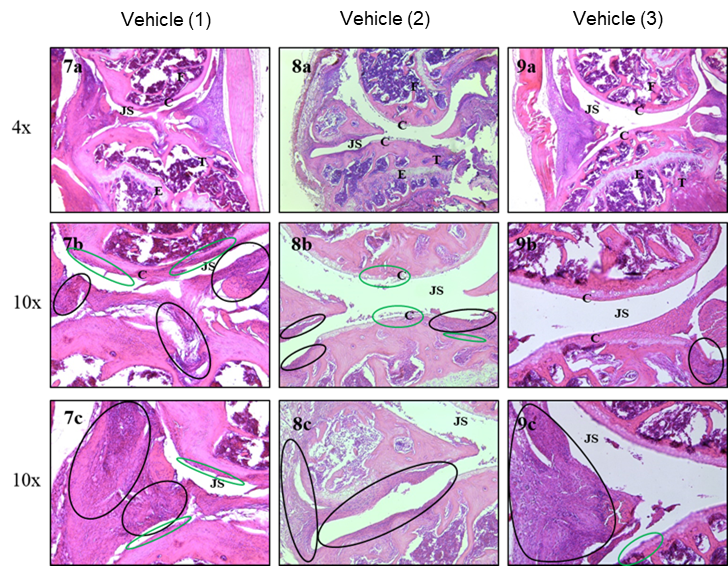


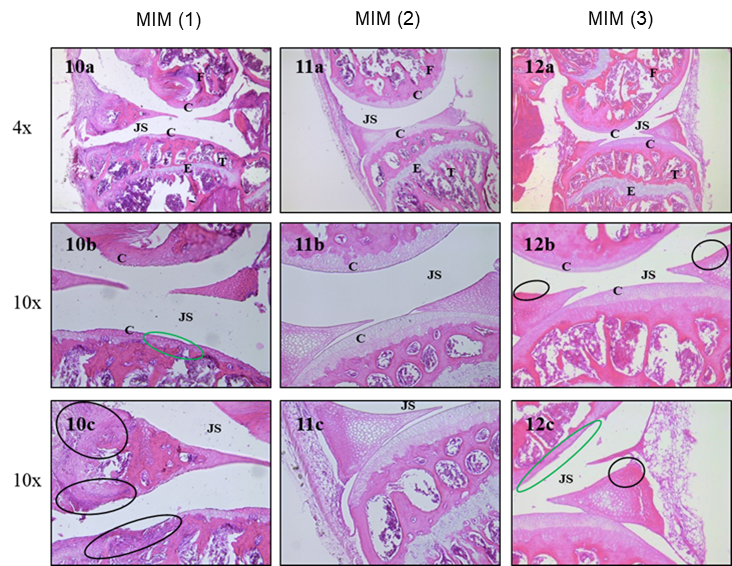


**Figure S1**

Representative histologic sections of knee joints of three animals per group stained with H&E from normal (1a-3c), CIA (4a-6c), vehicle (7a-9c) and MIM-treated mice (10a-12c) are shown. The magnification of the images in the first row was taken at 4x, whereas in the second and third row were taken at 10x. Black circles indicate inflammation and synovial hyperplasia. Green circles show articular cartilage loss. T=Tibia; F=Femur; C=Cartilage layer; JS=Joint space; E=Epiphyseal growth plate.


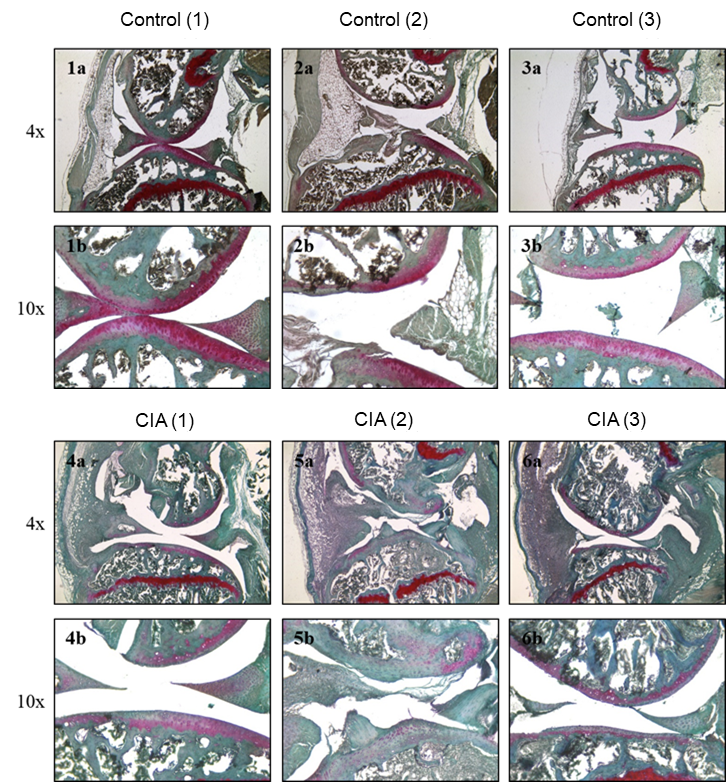


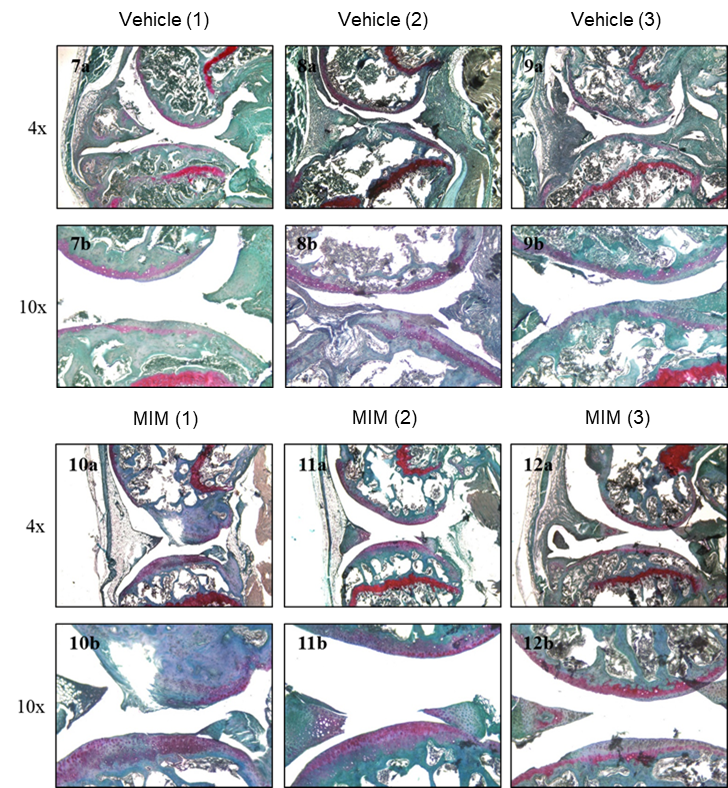


**Figure S2**

Representative histologic sections of knee joints of three animals per group stained with safranin-O from normal (1-3), CIA (4-6), placebo-treated (7-9) and 2LARTH-treated mice (10-12) are shown. The magnification of the images was taken at 4x and 10x. The areas with cartilage appear red colour stained.
